# Consanguinity among individuals with diabetes in Pakistan: A cross-sectional study

**DOI:** 10.1371/journal.pgph.0004964

**Published:** 2025-07-24

**Authors:** Anaya Abdul Samad, Safwat Irshad Qureshi, Ayesha Mukhtar Rathore, Azhan Ahmed, Warda Rasool, Samim Noori, Sardar Noman Qayyum, Muhammad Talha Kakar

**Affiliations:** 1 Department of Medicine, Bolan Medical College, Quetta, Balochistan, Pakistan; 2 Department of Medicine, Pakistan Institute of Medical Sciences, Islamabad, Pakistan; 3 Department of Medicine, King Edward Medical University, Lahore, Punjab, Pakistan; 4 Department of Medicine, Nangarhar University, Nangarhar, Afghanistan; 5 Department of Medicine, Bacha Khan Medical College, Mardan, Pakistan; Centre of Biomedical Ethics and Culture, PAKISTAN

## Abstract

To determine the frequency of consanguinity among individuals with diabetes in Pakistan and to investigate the effect of consanguinity on the occurrence of diabetes at different familial levels, we also aimed to report public perceptions on the matter. This cross-sectional study was conducted between August 2023 and January 2024, targeting individuals with diabetes across Pakistan. Data were collected through an online questionnaire, which included questions on participants’ demographics, family history, diabetes diagnosis, and awareness levels. Participants were classified based on their diabetes type. Data were analyzed using SPSS. Descriptive statistics were used to determine frequencies, and chi-square tests were applied to assess associations. Of the 404 participants, 52% reported having consanguineous parents, with 22.3% being first cousins. Type 2 diabetes was the most prevalent (70.5%), followed by Type 1 (26.5%) and gestational diabetes (3%). A strong family history of diabetes was reported by 80.4% of participants, with 41.1% having diabetic siblings. Around 64.8% demonstrated general knowledge about diabetes, and 63.1% agreed that consanguinity increases the risk of diabetes. The study reveals a high frequency of consanguineous parental relationships among individuals with diabetes in Pakistan. Public health interventions, including genetic counseling and awareness campaigns, are essential to address the risks associated with consanguineous marriages and reduce the diabetes burden in Pakistan.

## Introduction

Diabetes mellitus is a rapidly escalating global health concern, affecting an estimated 589 million adults aged 20–79 years, a figure projected to reach 853 million by 2050 [1]. The burden is disproportionately concentrated in low- and middle-income countries, where access to timely diagnosis and treatment is limited [1]. Pakistan faces a critical public health challenge, ranking fourth globally in terms of diabetes prevalence and first in the MENA region for age-standardized rates [2]. As of 2024, approximately 34.5 million adults in Pakistan are living with diabetes, with over 26.9% of cases remaining un-diagnosed and 226,000 deaths attributed to the disease annually [2,3]. Socioeconomic disparities, inadequate healthcare infrastructure, particularly in rural areas, and cultural practices amplify this epidemic, necessitating urgent, population-specific research to inform targeted interventions.

An important cultural factor contributing to genetic disease susceptibility in Pakistan is consanguinity, with approximately 63% of marriages involving close relatives, predominantly first cousins [4]. Consanguinity is defined as the act of marrying blood relatives [5]. This long-standing cultural norm significantly increases the likelihood of homozygosity for deleterious alleles, which may influence the expression and risk of various inherited disorders, including diabetes [6]. In addition to its genetic consequences, consanguinity is associated with increased rates of adverse pregnancy outcomes, congenital anomalies, and stillbirths [7,8].

Despite its profound implications for public health, research on the correlation between parental consanguinity and its associated diabetes risk in Pakistan remains scarce. Most existing literature addresses diabetes risk in general terms, without stratifying by diabetes type. This limits our understanding of whether consanguinity exerts a stronger influence on specific sub-types, such as type 1 diabetes mellitus (T1DM), type 2 diabetes mellitus (T2DM), or monogenic forms. There is a lack of empirical data examining how parental consanguinity contributes to the genetic risk of diabetes and whether public awareness of this association exists in Pakistan. These knowledge gaps impede the development of culturally and genetically informed prevention strategies. Without clarity on the relationship between consanguinity and diabetes sub-types, public health programs in high-risk populations like Pakistan lack the evidence needed to design effective screening, education, and genetic counseling initiatives.

Biologically, diabetes mellitus is not a uniform disease entity but encompasses distinct sub-types with different genetic mechanisms. T1DM is primarily autoimmune in origin, marked by T-cell–mediated destruction of pancreatic β-cells and associated with specific HLA genotypes [9]. Genetic susceptibility, autoimmunity and environmental triggers are important risk factors for Type 1 Diabetes Mellitus (T1DM) [10,11]. T2DM is driven by insulin resistance and β-cell dysfunction, underpinned by a polygenic architecture involving loci related to insulin signaling, glucose metabolism, and lipid regulation [12]. Obesity, sedentary lifestyle, metabolic conditions, demographics, family history and genetics are some of the well-known risk factors for type 2 Diabetes (T2DM) [13]. Monogenic forms of diabetes, such as maturity-onset diabetes of the young (MODY) and neonatal diabetes, result from rare, highly penetrant mutations in single genes that govern β-cell development, function, or insulin production [14]. Recessive inheritance patterns were observed in certain populations with a high prevalence of consanguinity, highlighting the importance of genetic context in diabetes subtyping. Monogenic diabetes arises from single-gene mutations (e.g., GCK, HNF1A) and is more likely to manifest in consanguineous populations due to increased homozygosity [15].

To our knowledge, this is the first study in Pakistan to assess the association between parental consanguinity and different types of diabetes, including T1DM, T2DM, and monogenic forms. The lack of such stratified data has limited the ability of public health programs to tailor genetic counselling, risk communication, and prevention strategies in culturally appropriate ways. This study fills a critical knowledge gap by clarifying whether consanguinity disproportionately influences specific diabetes sub-types in a population with high rates of intra-familial marriage. We aim to inform precision public health interventions and contribute to the development of targeted diabetes prevention and screening programs in consanguineous populations.

## Materials and methods

A cross-sectional survey design was employed to collect data from a representative sample of the Pakistani population. The questionnaire was adapted based on thematic structure from a previously published study by Alzahrani et al. (2021) in Saudi Arabia [13]. It was further refined through a pilot test using the first 20 responses to ensure clarity and usability. The questionnaire was designed to collect data on a range of demographic, familial, and health-related factors related to diabetes, with a particular focus on the potential impact of consanguinity. It included questions regarding the participant’s age, gender, residence, and region, as well as their personal history with diabetes, including the type of diabetes and the duration of the condition. The questionnaire also explored familial factors, such as the consanguinity of parents, family history of diabetes, and the occurrence of diabetes within extended family members. Participants were also asked about their awareness of diabetes, including its types, causes, and management strategies, and their perceptions of the role of consanguinity in increasing the risk of diabetes. The study focused solely on individuals with diabetes, comparisons were made between participants with consanguineous vs. non-consanguineous parental backgrounds to explore associations with diabetes-related characteristics. The design of the questionnaire aimed to capture both objective data and subjective beliefs, providing a comprehensive understanding of the various factors that may contribute to the development of diabetes within the context of consanguineous marriages.

The sample size was calculated using the OpenEpi software, based on a hypothesized frequency of 50% ± 5% for the outcome factor in the population, a confidence level of 95%, and a 5% margin of error. The estimated population size was set at 1,000,000, and a design effect of 1 was used for calculation. This yielded a minimum required sample size of 384 participants.

To ensure representativeness, the sample was stratified according to the population distribution of Pakistan’s provinces, including Punjab, Sindh, Khyber Pakhtunkhwa, Baluchistan, Islamabad, Gilgit-Baltistan, and Kashmir. Proportional allocation was used to assign the required number of participants from each province, reflecting their population sizes relative to the national census data. To ensure representation from rural populations, data collection was supplemented by field researchers who distributed the survey via mobile-assisted interviews in rural areas of Baluchistan, Interior Punjab, Interior Sindh and KPK. Data collection was carried out using an online questionnaire via Google forms for the ease of data processing.

A total of 412 individuals responded to the survey. After data cleaning, entries with incorrect or missing information were excluded, and all valid responses (404) were included in the analysis. Multiple trained data collectors were deployed for data collection, ensuring comprehensive coverage across the regions. Data collectors were provided with standardized protocols and guidelines to minimize variability and bias during the collection process. Eligibility criteria included participants of Pakistani origin, 18 years or older. Incorrect, incomplete or invalid responses were excluded from the study. Participants who selected “Don’t know” in response to parental consanguinity were excluded from analysis (4.2%), as accurate classification of exposure was not possible for this subgroup.

Data was exported to SPSS (statistical package for social sciences) version 27.0 for analysis. Descriptive statistics such as frequencies, percentages, and means were used to summarize participants’ characteristics and responses to survey items.

### Compliance with ethical standards

#### Research on humans.

The study conducted research on human beings following ethical standards. The study was approved by Institutional Review Board (IRB) of Bolan University of Medical Health Sciences, Quetta, prior to data collection (Ref No; BUMHS/IRB/23).

#### Informed consent.

All participants provided informed consent before participating in the study.

## Results

**Fig 1:**
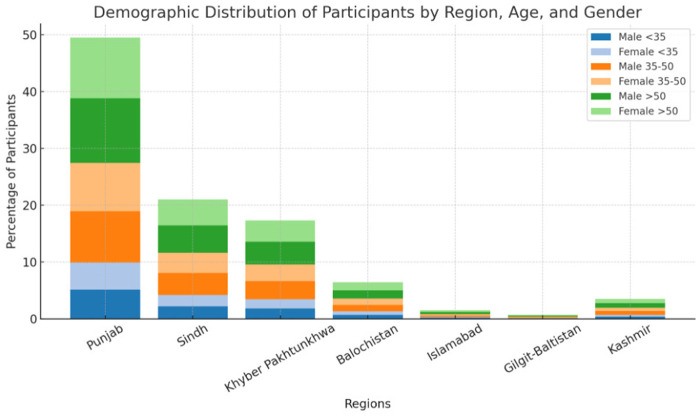
Demographic distribution of participants by region, age, and gender, showing the percentage breakdown across different provinces and territories in Pakistan.

A total of 404 valid responses were included in the analysis (Table 1). The age distribution of participants was such that, 44.6% of our participants were over 50 years old, 35.4% were between 35 and 50 years, and 20% were under 35 years. There were 51.5% male participants and 48.5% female participants. Geographically, the majority of participants were from Punjab (49.5%), followed by Sindh (21.0%) and Khyber Pakhtunkhwa (17.3%). Smaller proportions of participants were from Baluchistan (6.4%), Islamabad (1.5%), Gilgit-Baltistan (0.7%), and Kashmir (3.5%) (Fig 1)

**Table 1:** Frequency distribution of participant characteristics.

*Variables*		*Count (N)*	*N %*
**Age group**	Less than 35 years	81	20.00%
	Between 35 and 50 years	143	35.40%
	More than 50 years	180	44.60%
**Gender**	Male	208	51.50%
	Female	196	48.50%
**Province/Region**	Punjab	200	49.50%
	KPK	70	17.30%
	Sindh	85	21.00%
	Baluchistan	26	6.40%
	Islamabad	6	1.50%
	Gilgit Baltistan	3	0.70%
	Kashmir	14	3.50%
**Type of diabetes**	Type 1	107	26.50%
	Type 2	285	70.50%
	Gestational	12	3.00%
	None	0	0.00%
**Duration of diabetes**	Less than 1 year	45	11.10%
	1–5 years	162	40.10%
	6–10 years	110	27.20%
	More than 10 years	87	21.50%
	Not applicable	0	0.00%
**Parents consanguinity**	Yes	210	52.00%
	No	177	43.80%
	I don’t know	17	4.20%
**Consanguinity Type**	1st cousin	90	22.30%
	2nd/3rd cousin	85	21.00%
	Common 4th-7th grand parents	38	9.40%
	Non-consanguineous	191	47.30%
**Parents diabetes status**	Father	85	21.00%
	Mother	133	32.90%
	Both	66	16.30%
	None	120	29.70%
**Parents diabetes type**	Type 1	41	10.10%
	Type 2	206	51.00%
	Mother type 1 father type 2	3	0.70%
	Father type 1 mother type 2	7	1.70%
	Both type 1	6	1.50%
	Both type 2	26	6.40%
	None	115	28.50%
**Paternal or maternal side diabetes**	Yes	325	80.40%
	No	79	19.60%
**Family Diabetes Prevalence**	Siblings	166	41.10%
	1st/2nd cousin	40	9.90%
	Uncle/Aunt	75	18.60%
	Distant relatives	74	18.30%
	None	49	12.10%
**Common diabetes in family**	Type 1	78	19.30%
	Type 2	240	59.40%
	I don’t know	86	21.30%

Type 2 diabetes was the most frequently reported (70.5%), followed by Type 1 (26.5%) and gestational diabetes (3%). The duration of diabetes varied, with 40.1% of participants having lived with the condition for 1–5 years, 27.2% for 6–10 years, and 21.5% for more than 10 years (Table 1).

52% of participants reported that their parents were consanguineous, with 22.3% indicating that their parents were 1st cousins (Table 1). 80.4% of participants reported a family history of diabetes, with 41.1% mentioning diabetes in their siblings. Type 2 diabetes (T2DM) was the most prevalent (70.5%), followed by Type 1 diabetes (T1DM, 26.5%) and gestational diabetes (3%) (Table 1). Due to its low prevalence, gestational diabetes was excluded from association analyses but included in descriptive statistics. Around 32.9% of participants reported that their mothers had diabetes, 21% reported their fathers had diabetes, and 16.3% reported both parents had diabetes. Type 2 diabetes was the predominant form of diabetes reported in both parents (51.0%). A higher proportion of participants with Type 1 diabetes reported parental consanguinity (60.7%) compared to those with Type 2 (48.1%), this association was not statistically significant (p = 0.144).

While T2DM was the most reported diabetes sub-type in our sample (70.5%), associations with consanguinity were strongest among those reporting T1DM and early-age diagnosis, aligning more closely with mechanisms of recessive inheritance. These findings do not imply uniform genetic susceptibility across all diabetes types but highlight the potential role of consanguinity in genetically driven forms.

The survey revealed substantial awareness of diabetes and its genetic risk factors among participants. The majority of participants (64.8%) reported having knowledge about diabetes and its causes. A similar proportion (63.1%) agreed that consanguinity increases the chances of diabetes development. (Table 2).

**Table 2:** Perception about consanguinity affiliated diabetes risk.

Variable	N	% Of total (N = 404)
***I have knowledge about diabetes, its causes, types and how to deal with it***		
Strongly agree	74	18.3
Agree	188	46.5
I don’t know	121	30
Disagree	18	4.5
Strongly disagree	3	0.7
***Genetic factors play an important role in development of diabetes***		
Strongly agree	152	37.6
Agree	164	40.6
I don’t know	79	19.6
Disagree	9	2.2
Strongly disagree	0	0.0
***Consanguinity increases chances of diabetes development***		
Strongly agree	89	22.0
Agree	166	41.1
I don’t know	122	30.2
Disagree	87	6.7
Strongly disagree	0	0.0

To address potential confounding by age, gender, and geographic region, stratified analyses were conducted which examined the distribution of parental consanguinity and diabetes types (T1DM, T2DM, gestational) across these variables (Table 3). Among 404 participants (excluding 17 “Don’t know” responses for consanguinity, 4.2%), consanguinity prevalence varied significantly by age (Chi-square p = 0.000), with 63.0% of participants aged <35 years reporting consanguineous parents compared to 45.6% in those >50 years. T1DM was more prevalent in younger participants (55.6% in <35 years vs. 18.3% in >50 years), while T2DM dominated in older groups (81.7% in >50 years vs. 37.0% in <35 years; Chi-square p = 0.000). Consanguinity differed by locality (p = 0.027), with urban participants reporting higher rates (56.0% vs. 41.6% rural), and T1DM was more common in rural areas (33.6% vs. 23.7%; p = 0.053). Gender showed no significant consanguinity difference (50.5% male vs. 53.6% female, p = 0.263), but diabetes types varied significantly (p = 0.001), with gestational diabetes exclusive to females (6.1%). Provincial differences in consanguinity were not significant (p = 0.183), though rates ranged from 28.6% (Kashmir) to 57.7% (Baluchistan), and diabetes types differed significantly (p = 0.042), with T1DM absent in Gilgit-Baltistan and Kashmir (Table 4).

**Table 3:** Stratified distribution of consanguinity and diabetes types across demographic characteristics.

Demographic Characteristic	Stratum	n (Total = 404)	% Consanguineous	% Non- Consanguineous	%T1DM	%T2DM	%Gestational
**Age group** **(p = 0.001)**	***<35 years***	81	63.0%	37.0%	55.6%	37.0%	7.4%
	***35–50 years***	143	53.8%	45.5%	20.3%	75.5%	4.2%
	***>50 years***	180	45.6%	45.6%	18.3%	81.7%	0.0%
**Locality Type** **(p = 0.027)**	***Urban***	291	56.0%	40.5%	23.7%	72.5%	3.8%
	***Rural***	113	41.6%	52.2%	33.6%	65.5%	0.9%
**Gender** **(p = 0.263)**	***Male***	208	50.5%	43.8%	26.0%	74.0%	0.0%
	***Female***	196	53.6%	43.9%	27.0%	66.8%	6.1%
**Province** **(p = 0.183)**	***Punjab***	200	56.5%	38.5%	25.0%	71.0%	4.0%
	***KPK***	70	38.6%	54.3%	40.0%	58.6%	1.4%
	***Sindh***	85	55.3%	44.7%	18.8%	77.6%	3.5%
	***Baluchistan***	26	57.7%	38.5%	38.5%	61.5%	0.0%
	***Islamabad***	6	50.0%	50.0%	50.0%	50.0%	0.0%
	***Gilgit Baltistan***	3	33.3%	66.7%	0.0%	100.0%	0.0%
	***Kashmir***	14	28.6%	64.3%	0.0%	100.0%	0.0%

**Table 4:** Chi-Square P-values for associations of parental consanguinity and diabetes types with demographic and geographic characteristics.

Demographic Characteristic	Parental Consanguinity (Yes/No) (P- value)	Diabetes Types (T1DM/T2DM/Gestational) (P- value)
***Age Group***	0.000*	0.000*
***Gender***	0.263	0.001*
***Locality Type***	0.027*	0.053
***Province***	0.183	0.042*

*Statistically significant at p < 0.05.

## Discussion

This study provides critical insights into the frequency of parental consanguinity and its association with diabetes risk in Pakistan. The findings reveal that 52% of participants reported consanguineous parents, with 22.3% being first cousins, highlighting the widespread practice of consanguinity in the country. The high prevalence of Type 2 diabetes (70.5%) among participants, coupled with a strong family history of diabetes (80.4%), highlights the significant role of genetic and familial factors in diabetes development. These results align with previous studies that have identified consanguinity as a risk factor for non-communicable diseases, including diabetes, particularly in populations with high rates of consanguineous marriages [16].

Our study observed a higher frequency of consanguinity among individuals with Type 2 diabetes, though this association was not statistically significant. This numerical trend aligns with findings from Saudi Arabia [17]. The increased risk of diabetes in individuals with consanguineous parents can be attributed to the higher likelihood of inheriting similar alleles at loci associated with diabetes susceptibility [18]. This is further supported by the observation that 41.1% of participants reported diabetes in their siblings, suggesting a strong genetic component. However, the lack of a significant association between consanguinity and Type 1 diabetes aligns with previous studies, such as those conducted in Saudi Arabia, which found no clear link between consanguinity and Type 1 diabetes [19].

Despite the high frequency of parental consanguinity and diabetes, the study revealed a notable awareness gap regarding the genetic risks associated with consanguinity. Awareness about diabetes was moderate, with 64.8% of participants reporting knowledge about its causes and types. Similarly, 63.1% believed that consanguinity increases the risk of developing diabetes, showing that while general knowledge about diabetes is relatively high, targeted awareness campaigns are needed to educate the public about the genetic risks posed by consanguineous marriages. Interestingly, participants with a family history of diabetes on either the maternal or paternal side were less likely to attribute diabetes to genetic factors, possibly due to the influence of lifestyle and environmental factors, such as diet and physical inactivity, which are well-documented contributors to Type 2 diabetes [17,18,20].

While this study did not directly assess genetic markers, the observed association between consanguinity and diabetes, especially among younger individuals and those reporting T1DM, may be consistent with biological mechanisms involving recessive inheritance and HLA homozygosity. However, given the reliance on self-reported diagnoses and the absence of genetic or lifestyle data, potential confounding from shared familial environments (e.g., diet, access to care, sedentary behavior) cannot be excluded. Future studies integrating genetic testing and lifestyle assessment are necessary to disentangle these overlapping influences.

The persistence of consanguineous marriages in Pakistan, despite increasing literacy rates and awareness of their health risks, raises important sociocultural questions. Consanguinity is deeply rooted in cultural and economic practices, particularly in rural areas, where it is often seen as a means of strengthening family ties and preserving wealth [21–23]. However, this practice has significant public health implications, as it not only increases the risk of diabetes but also contributes to a higher burden of congenital anomalies, developmental disorders, and other non-communicable diseases [24,25]. Given the high prevalence of consanguineous marriages in Pakistan, public health strategies must consider culturally sensitive approaches to genetic counseling. Rather than discouraging consanguinity outright, which may be culturally unacceptable and impractical, efforts should focus on increasing community awareness of the potential genetic risks associated with intra-familial unions, particularly for disorders like Type 1 diabetes and monogenic diabetes. Integration of genetic counseling services into existing healthcare frameworks, including maternal and child health programs, primary care clinics, and premarital counseling initiatives, could be an effective strategy. Utilizing religious leaders, local health workers, and community-based platforms may also help overcome stigma and resistance, fostering informed decision-making while respecting cultural norms. Addressing this issue requires genetic counseling, public health education, and policy interventions aimed at reducing the prevalence of consanguineous marriages [26]. Our findings support the need for public health strategies such as genetic counseling, especially in high-consanguinity regions of Pakistan. Community-level awareness campaigns, preferably conducted in local languages, can play a positive role in reducing genetic risk factors associated with diabetes in Pakistan. One of the key strengths of this study is its focus on the Pakistani population, where both diabetes and consanguinity are highly prevalent but understudied [27]. By examining the connections between these factors, the study provides valuable insights for public health planning and intervention.

### Limitations

The study did not include a control group of non-diabetic individuals, which limits our ability to assess whether consanguinity increases the risk of developing diabetes in the general population. Instead, internal comparisons were made between diabetic individuals with and without parental consanguinity to explore associations. Additionally, our study did not collect information on comorbidities or physical deformities in participants, which may have offered further insights into genetic implications of consanguinity. The lack of statistically significant findings between consanguinity and diabetes type can be attributed to insufficient power. The cross-sectional design limits the ability to establish causal relationships. Reliance on self-reported data may introduce recall bias, particularly regarding family history and consanguinity status. No clinical verification or genetic testing was conducted to validate diabetes types or consanguinity status, which may have introduced mis-classification bias. The study also did not control for important confounders such as BMI, socioeconomic status, and physical activity, which limits the ability to isolate genetic associations.

Future studies should consider longitudinal designs, larger sample size, non-diabetic comparative groups to better understand the temporal relationship between consanguinity and diabetes development, as well as epidemiological studies to quantify the relative contributions of genetic, lifestyle, and environmental factors [28].

## Conclusion

This cross-sectional study highlights the high frequency of parental consanguinity and diabetes in Pakistan, shedding light on the potential interplay between genetic and familial factors in diabetes risk. The findings reveal that 52% of participants had consanguineous parents, with 22.3% being first cousins, reflecting the deep-rooted cultural practice of consanguinity in the country. The high prevalence of Type 2 diabetes (70.5%) and a strong family history of diabetes (80.4%) underscore the need for further research to explore the genetic and environmental determinants of diabetes in this population. While the study does not establish causality, the observed patterns align with existing literature suggesting that consanguinity may contribute to an increased risk of non-communicable diseases, including diabetes. Public health interventions, such as genetic counseling and awareness campaigns, are urgently needed to educate the population about the potential risks associated with consanguineous marriages. Addressing these issues is critical for reducing the burden of diabetes and improving health outcomes in Pakistan.
